# The Use of ProteoTuner Technology to Study Nuclear Factor κB Activation by Heavy Ions

**DOI:** 10.3390/ijms222413530

**Published:** 2021-12-16

**Authors:** Arif Ali Chishti, Christa Baumstark-Khan, Hasan Nisar, Yueyuan Hu, Bikash Konda, Bernd Henschenmacher, Luis F. Spitta, Claudia Schmitz, Sebastian Feles, Christine E. Hellweg

**Affiliations:** 1 Radiation Biology, Institute of Aerospace Medicine, German Aerospace Center (DLR), Linder Höhe, D-51147 Köln, Germany; arifalichishti@szu.edu.cn (A.A.C.); christa.baumstark-khan@dlr.de (C.B.-K.); hasan.nizar@dlr.de (H.N.); yueyuan.hu@gmail.com (Y.H.); bikash.konda@dlr.de (B.K.); bhenschenmacher@bfs.de (B.H.); luis.spitta@dlr.de (L.F.S.); claudia.schmitz@dlr.de (C.S.); sebastian.feles@dlr.de (S.F.); 2 Department of Biochemistry and Molecular Biology, School of Medicine, Shenzhen University, Shenzhen 518055, China; 3 Department of Medical Sciences, Pakistan Institute of Engineering and Applied Sciences (PIEAS), Lehtrar Road, Nilore, Islamabad 45650, Pakistan; 4 Institute of Cardiovascular Immunology, University Hospital Bonn, D-53127 Bonn, Germany; 5 Competence Center for Electromagnetic Fields (KEMF), Federal Office for Radiation Protection, Ingolstädter Landstraße 1, D-85764 Oberschleißheim, Germany; 6 Gravitational Biology, Institute of Aerospace Medicine, German Aerospace Center (DLR), Linder Höhe, D-51147 Köln, Germany

**Keywords:** nuclear factor κB, reporter system, ProteoTuner system, heavy ions, X-rays, tdTomato, DD-tdTomato, fluorescent protein, galactic cosmic rays, space missions

## Abstract

Nuclear factor κB (NF-κB) activation might be central to heavy ion-induced detrimental processes such as cancer promotion and progression and sustained inflammatory responses. A sensitive detection system is crucial to better understand its involvement in these processes. Therefore, a DD-tdTomato fluorescent protein-based reporter system was previously constructed with human embryonic kidney (HEK) cells expressing DD-tdTomato as a reporter under the control of a promoter containing NF-κB binding sites (HEK-pNFκB-DD-tdTomato-C8). Using this reporter cell line, NF-κB activation after exposure to different energetic heavy ions (^16^O, 95 MeV/n, linear energy transfer—LET 51 keV/µm; ^12^C, 95 MeV/n, LET 73 keV/μm; ^36^Ar, 95 MeV/n, LET 272 keV/µm) was quantified considering the dose and number of heavy ions hits per cell nucleus that double NF-κB-dependent DD-tdTomato expression. Approximately 44 hits of ^16^O ions and ≈45 hits of ^12^C ions per cell nucleus were required to double the NF-κB-dependent DD-tdTomato expression, whereas only ≈3 hits of ^36^Ar ions were sufficient. In the presence of Shield-1, a synthetic molecule that stabilizes DD-tdTomato, even a single particle hit of ^36^Ar ions doubled NF-κB-dependent DD-tdTomato expression. In conclusion, stabilization of the reporter protein can increase the sensitivity for NF-κB activation detection by a factor of three, allowing the detection of single particle hits’ effects.

## 1. Introduction

During space missions, astronauts are chronically exposed to galactic cosmic rays at a low dose rate. Heavy ions make up only about 2% of the fluence in space. However, payload limitations in spacecraft design, the production of secondary particles in shielding material, and long durations of deep space travel impede in completely removing the probability of exposure to such heavy ions. Additionally, heavy ions are also gaining wider popularity for clinical use to effectively target and treat solid malignancies. Therefore, further study of the biological effects of heavy ions is vital to improve the health effects estimation in humans as the canvas of space flight spreads to include larger populations of people as well as to better predict acute and long-term effects of their use in a clinical setup.

Heavy ions, similarly to all ionizing radiations, stimulate multiple stress-inducible signaling pathways that can activate multiple transcription factors and thus alter the expression of multiple target genes [1]. The members of the nuclear factor κB (NF-κB) family are tightly regulated transcription factors that are usually activated by cytokines and also by a genotoxic stress-induced pathway after ionizing radiation exposure and play a pivotal role in the cellular response to DNA damage [2,3]. In case of radiation exposure, NF-κB on one hand improves cellular survival to escape from lethal effects of DNA damage; on the other hand, it increases cancer risk and contributes to chemo- and radio-resistance [4,5,6]. NF-κB pathway activation depends on the stimulus, cell type, and physical parameters of ionizing radiation including quality of radiation, dose, or fluence.

The quality of radiation can be described as the average energy transferred by an ionizing particle when it passes through matter and is known as linear energy transfer (LET) [7]. The absorbed dose is the energy deposited in Joules per kilogram mass of irradiated matter. The fluence describes the number of particles that traverse a unit area and is usually given in particles per cm^2^ (P/cm^2^). The dose and fluence required to produce a particular biological effect within human cells depend on the LET of incident heavy ions. In the range up to ≈200 keV/µm, the higher the LET, the lower the dose and fluence needed for a similar killing effect. At very high LET values (>200 keV/µm), an overkill effect is observed [8].

**Figure 1:**
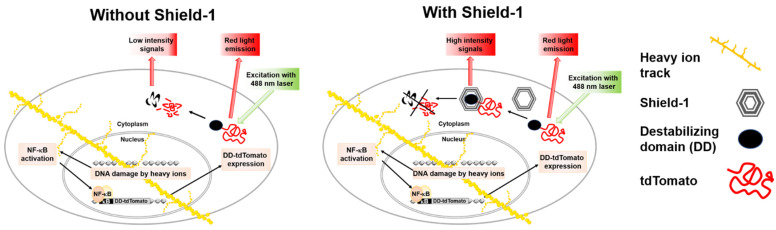
Principle of the HEK-pNFκB-DD-tdTomato-C8 cells as reporter assay for NF-κB activation. HEK cells were stably transfected with the plasmid pNFκB-DD-tdTomato containing four κB sites as binding sites for the transcription factor NF-κB, which control the expression of the reporter protein DD-tdTomato. A heavy ion passing through the cell leads to a multitude of ionizations along its track (indicated as big yellow dots), and it produces secondary electrons that can give rise to ionizations outside of the primary particle track (small yellow dots). These ionizations can induce DNA double-strand breaks which initiate the activation of NF-κB via the genotoxic stress-induced NF-κB subpathway. Activated NF-κB enters the cell nucleus and binds to κB sites in promoters, including the κB sites that control DD-tdTomato expression. The DD-tdTomato mRNA travels to the cytoplasm and is translated to the DD-tdTomato protein, whose red fluorescence can be detected after correct protein folding. DD-tdTomato is rapidly degraded in the absence of Shield-1 due to the destabilizing domain (DD). In the presence of Shield-1, DD-tdTomato is stabilized and accumulates in the cytoplasm, leading to a higher red fluorescence signal after excitation with the 488 nm argon laser of the flow cytometer.

To determine the LET dependence of NF-κB activation by heavy ions, sensitive reporter systems can be used in human cell lines. The discovery of fluorescent proteins has greatly simplified the understanding of complex signaling pathways. Reporter systems based on fluorescent proteins represent a powerful approach for fast and convenient gene regulation studies in radiation research. The sensitivity of fluorescent protein-based reporter systems mainly depends on the intensity and stability of reporter signals and linked promoter inducibility [9]. In imaging experiments, it is also considered that the reporter fluorescent protein has to be bright, photostable, insensitive to environmental effects, and non-toxic in the chosen system to avoid confounders in the quantitative interpretation of experimental results. The control of fluorescent protein levels enabled by ProteoTuner systems opens up new possibilities in radiation research. The ProteoTuner system is based on the mutant L106P of a protein with the abbreviated name FKBP12, which means tacrolimus (FK506) binding protein, 12 kilodalton molecular weight (FKBP12) protein, and a fused fluorescent protein. The human FKBP12 protein is the parent molecule, and the mutant L106P of FKBP12 (12 kDa) is a destabilizing domain (DD), which is rapidly degraded in mammalian cells. For real-time measurement of transcription factor activation, a fluorescent protein with a very short half-life but with high brightness is preferable in the ProteoTuner system. The tandem dimer (td) variant of the red fluorescent protein Tomato is up to six times brighter than Enhanced Green Fluorescent Protein (EGFP) and provides strong real-time signals when tagged with the DD L106P. The ligand, Shield-1, is a small synthetic membrane permeable molecule (750 Da) that has very high affinity to bind to L106P, and its addition stabilizes the L106P and tdTomato fusion protein in a reversible and dose-dependent manner. The ProteoTuner system has recently been used to regulate the expression of the proteins Mitogen-activated protein kinase phosphatase 3 (MKP3), SRY (sex determining region Y)-box transcription factor 1 (SOX1), and the pluripotency regulator PR/SET domain 14 (PRDM14) [10,11,12]. Previously, a Human Embryonic Kidney (HEK/293) cell line-based reporter system, HEK-pNFκB-DD-tdTomato-C8, was constructed using the ProteoTuner system to evaluate NF-κB signaling in living cells after low LET radiation (X-rays) exposure [9]. This reporter system serves as a simple screening assay for quick and reliable measurements of cellular NF-κB signal transduction through the expression of the DD-tdTomato reporter gene secondary to NF-κB activation (Figure 1).

It was successfully applied to study the kinetics and dose response of NF-κB activation after exposure to camptothecin (CPT), tumor necrosis factor α (TNF-α), and X-rays [9]. In the current study, the HEK-pNFκB-DD-tdTomato cell line was used to define the LET- and fluence-dependence of NF-κB activation in response to heavy ions of different LET values. Furthermore, the lowest fluence that activates NF-κB was calculated.

## 2. Results

The activation of NF-κB by three different heavy ions in comparison to X-rays was determined by means of the reporter cell line HEK-pNFκB-DD-tdTomato-C8, revealing a strong dose, fluence, and LET dependency of its activation. Stabilization of the rapidly degraded DD-tdTomato using the synthetic molecule Shield-1 resulted in a higher sensitivity of the reporter system.

### 2.1. NF-κB-Dependent DD-tdTomato Expression after Exposure to Heavy Ions

**Figure 2:**
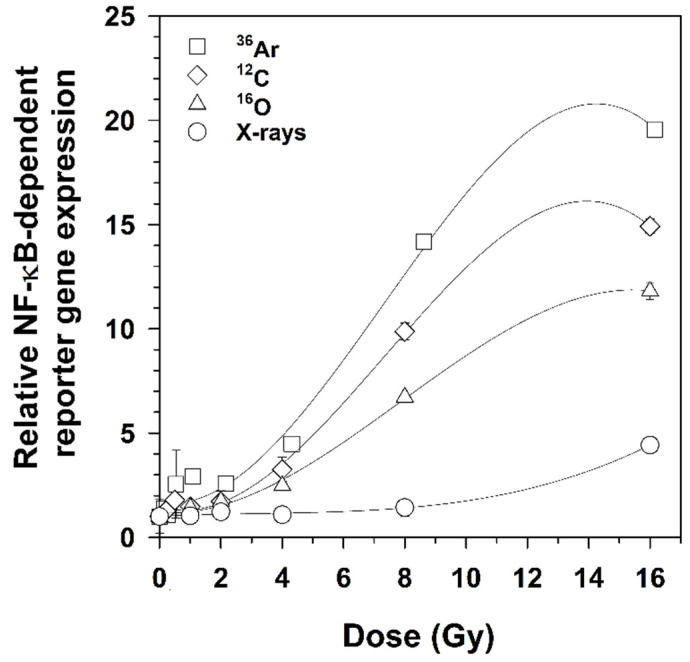
Activation of NF-κB-dependent DD-tdTomato expression. Cells were fixed 18 h after exposure to ^36^Ar (LET 272 keV/µm), ^12^C (LET 73 keV/μm), ^16^O (LET 51 keV/µm) ions, or X-rays. DD-tdTomato fluorescence intensity of the cells was measured by flow cytometry. The percentage of DD-tdTomato(+) cells in irradiated cell populations was normalized to that percentage in the mock-irradiated samples (0 Gy) (Mean ± SD, *n* = 2). Where the error bars are not visible, they are smaller than the symbol.

HEK-pNFκB-DD-tdTomato-C8 reporter cells were exposed to heavy ions or X-rays, as shown in Table 1. Previous experiments with HEK-pNFκB-DD-tdTomato-C8 cells revealed that the maximal DD-tdTomato fluorescence occurred 16–18 h after irradiation [9]. Therefore, 18 h was chosen as the optimum time point for measuring DD-tdTomato expression after X-rays and heavy ion exposure. Fluence was calculated from the Poisson distribution of heavy ion hits of cell nuclei, and dose was calculated as mentioned in Section 4.7.1. It was observed that NF-κB-dependent DD-tdTomato expression was much higher after heavy ions exposure compared to X-rays at higher doses (Figure 2). A clear difference in NF-κB activation was observed after 4 Gy of high- and low-LET radiation qualities. Among these different radiation qualities, NF-κB was activated by ^36^Ar ions to a higher extent than by ^12^C ions, ^16^O ions, and X-rays. The activation of NF-κB did not follow a linear pattern above 8 Gy due to the increasing damaging capacity of heavy ions. Average doses that double the NF-κB-dependent DD-tdTomato expression after exposure to different heavy ions were also calculated and listed in Table 1.

### 2.2. Effect of Fluence on NF-κB-Dependent DD-tdTomato Expression

**Figure 3:**
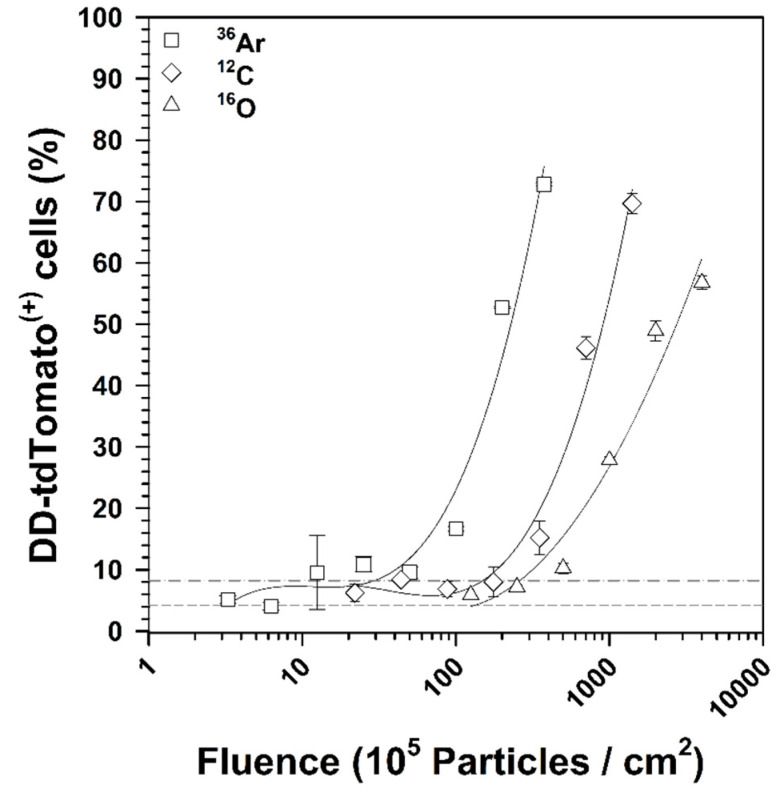
NF-κB activation in response to different heavy ion fluences. HEK-pNFκB-DD-tdTomato-C8 cells were fixed 18 h after exposure to ^36^Ar (LET 272 keV/µm), ^12^C (LET 73 keV/μm), and ^16^O (LET 51 keV/µm) ions. DD-tdTomato expression was determined by measuring the fluorescence intensity using flow cytometric analysis. The percentage of DD-tdTomato positive cells (DD-tdTomato (+)) was determined using histograms of the red fluorescence (see Supplementary Figure S1) and is shown as mean ± SD (*n* = 2). The short dash line indicates the percentage of DD-tdTomato(+) cells in mock-irradiated samples and the dash–dot line indicates the double of this percentage, which was defined as the threshold for NF-κB activation.

It was also observed that both LET and fluence affect the induction of NF-κB-dependent DD-tdTomato expression in HEK cells, which is represented as percentage of DD-tdTomato(+) cells in Figure 3. Flow cytometric analysis revealed a very strong fluorescence increase in this subpopulation that was dose-dependent (Supplementary Figure S1). The Poisson distribution of the heavy ion hits in the cell nuclei was calculated according to Equation (3). Based on this, 44 hits of ^16^O ions and 45 hits of ^12^C ions per cell nucleus were required to double the NF-κB-dependent DD-tdTomato expression, whereas 3 hits of ^36^Ar (95 MeV/n, LET 272 keV/µm) were sufficient to have the similar effect (2 × NF-κB activation). The relative biological effectiveness for NF-κB activation (RBE_NF-κB_) was also calculated, as mentioned in Section 4.7.3, and this is listed in Table 1.

### 2.3. Effect of Shield-1 on NF-κB-Dependent DD-tdTomato Expression after Heavy Ion Exposure

**Figure 4:**
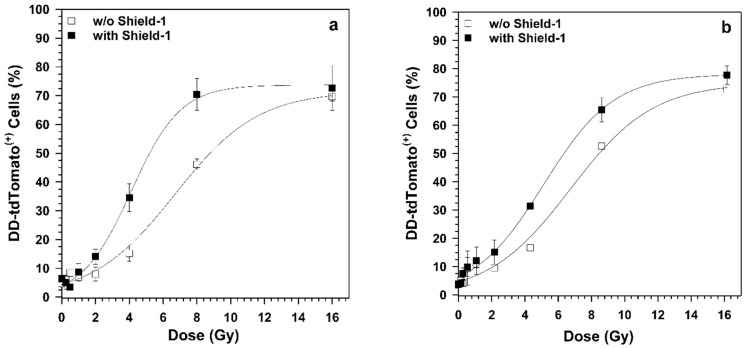
Effect of Shield-1 on NF-κB-dependent DD-tdTomato expression. HEK-pNFκB-DD-tdTomato-C8 cells were fixed 18 h after exposure to (**a**) ^12^C ions (LET 51 keV/µm) and (**b**) ^36^Ar ions (LET 272 keV/µm) in the absence and presence of Shield-1. NF-κB-dependent DD-tdTomato expression was determined using flow cytometric analysis. The percentage of DD-tdTomato positive cells (DD-tdTomato(+)) was determined from the red fluorescence histograms (see Supplementary Figure S1) and plotted as mean ± SD (*n* = 2).

The dose–effect relationships of NF-κB-dependent DD-tdTomato expression after exposure to ^12^C and ^36^Ar ions in the presence or absence (*w/o* = without) of Shield-1 were analyzed by quantifying the percentage of DD-tdTomato positive cells from flow cytometry histograms, as shown in Figure 4. Both ions strongly increased DD-tdTomato fluorescence dose-dependently, indicating the activation of the NF-κB pathway. This increase of DD-tdTomato(+) cells occurred at low doses when Shield-1 was added to allow a higher accumulation of DD-tdTomato.

### 2.4. Induction of NF-κB-Dependent DD-tdTomato Expression by a Single Particle Hit

Doses initiating the duplication of NF-κB-dependent DD-tdTomato fluorescence were derived from a regression curve of NF-κB activation versus dose (as shown in Figure 4), where the 0 Gy sample was set as the threshold. The average dose and heavy ions hits per nucleus that double the NF-κB-dependent DD-tdTomato expression in the presence or absence of Shield-1 are listed in Table 1. When a comparison of the accumulated signal and near real-time signals was done—by comparing DD-tdTomato(+) cells in the presence or absence of Shield-1—it was found out that even a single particle hit of an ^36^Ar ion (95 MeV/n, LET 272 keV/µm) can double the NF-κB-dependent DD-tdTomato expression.

### 2.5. Fold Induction of NF-κB-Dependent Promoter Activity

**Figure 5:**
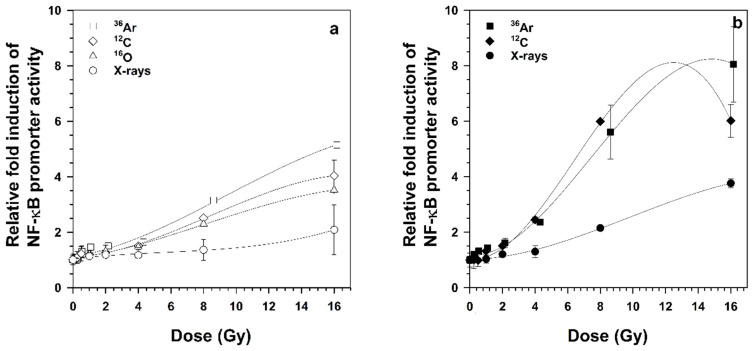
Relative fold induction of NF-κB promoter activity. Cells were fixed 18 h after exposure to ^36^Ar ions (LET 272 keV/µm), ^12^C ions (LET 73 keV/μm), and ^16^O ions (LET 51 keV/µm), and X-rays in the absence (**a**) or presence (**b**) of Shield-1. The fluorescence of NFκB-DD-tdTomato cells was analyzed using flow cytometry. The relative fold induction of NF-κB promoter activity was calculated by dividing the geometrical mean of fluorescence intensity (MFI) of the irradiated cells by the MFI of the non-irradiated control. Mean ± SD (*n* = 2) are shown.

HEK-pNFκB-DD-tdTomato-C8 cells are a dual reporter system that can provide both discrete and cumulative signals. In the presence of Shield-1, the fluorescent protein DD-tdTomato is not degraded but accumulated inside the cell, which helps to quantify the fold induction of NF-κB-promoter activity (Figure 5). The geometrical mean of fluorescence intensity (MFI) of the gated population (intact cells after removal of debris) was measured after exposure to heavy ions and X-rays. Fold up-regulation of NF-κB promoter activity was calculated by Equation (4). In the presence of Shield-1, the fluorescent protein DD-tdTomato was higher compared to cells that were incubated without Shield-1 after irradiation (Figure 5). In the absence of Shield-1, the relative promoter activity after ^16^O ion exposure was comparable to the activity after ^12^C ion exposure. Figure 5b shows that the promoter activity after exposure to heavy ions of the same energy followed a similar pattern with a strong dose-dependent increase up to 8 Gy and trend to saturation at higher doses. The dose-dependent increase was much flatter after irradiation with X-rays.

## 3. Discussion

An inducible promoter can be used for examination of the gene expression changes in specific pathways in response to different agents including ionizing radiation; a constitutively active promoter is suitable for use when detecting cytotoxicity [13]. A reporter gene associated with a regulatory sequence, promoters, enhancers or repressor binding sequences can be used to study gene expression. Stable transfection of the reporter construct into the target cell line eliminates the need for transient transfection and thereby reduces inter-assay variability. For reporter proteins not requiring a substrate, e.g., fluorescent proteins with a long half-life and high stability, considerable background fluorescence can accumulate and thereby reduce the dynamic range of the promoter–reporter assay. Reducing the half-life of the fluorescent protein by fusion with a destabilizing domain helps to reduce the accumulated background fluorescence [14], but it also limits the maximal fluorescence signal that can be reached after promoter activation. The ProteoTuner system, which is based on a destabilizing domain (DD) sequence linked to the tdTomato sequence and a small molecule called Shield-1 to stabilize DD-tdTomato, allows reduction of the background fluorescence of the stably transfected reporter cell line in combination with high fluorescence signals by stabilization of DD-tdTomato when Shield-1 is added after or during promoter activation by different agents. The suitability of DD-tdTomato as a transcription reporter has previously been tested [9]. Therefore, the Pro-teoTuner technology represents a powerful approach for fast and convenient gene regulation studies. By using this technology, a DD-tdTomato reporter system for NF-κB activation was constructed previously and tested with X-rays for the screening of NF-κB activation [9]. In this cell line, an inducible promoter containing NF-κB binding sites was linked with DD-tdTomato. Thus, the fluorescence intensity of DD-tdTomato can be used to directly correlate gene induction to calculate the fold induction of the linked promoter.

The activation of NF-κB can be initiated via canonical, alternative, or genotoxic stress-induced pathways [15]. In the canonical pathway, activation is triggered via binding of receptor ligands such as pro-inflammatory cytokines e.g., tumor necrosis factor α (TNF-α) or interleukin 1 (IL-1), growth factors, or pathogen-associated molecular patterns (PAMPs), to membrane receptors (cytokine receptors, Toll-like receptors, TLRs). This pathway plays a critical role during inflammation, and NF-κB activation occurs quickly [15]. The alternative pathway is also mediated through membrane receptors, but it is involved in non-inflammatory signaling. The genotoxic stress-induced pathway is initiated by DNA double-strand breaks in the cell nucleus. Ionizing radiation-induced DNA double-strand breaks trigger this kind of atypical NF-κB activation pathway that involves the serine/threonine kinase ataxia telangiectasia mutated (ATM), which is activated in response to DNA double-strand breaks [15]. In addition to DNA double-strand breaks that are induced by the physical agent ionizing radiation, breaks induced by chemical agents such as the topoisomerase inhibitor camptothecin or reactive oxygen species (ROS) can activate this NF-κB subpathway [8,9,16,17]. The amount and complexity of DNA double-strand breaks depend on the quality of radiation (e.g., X-ray, α, β, γ radiation or heavy ions). High-LET radiation such as heavy ions are known to generate complex damage, which is difficult to repair accurately and completely and therefore have a higher efficiency in activating NF-κB compared to X-rays [17]. In this study, we used the DD-tdTomato reporter construct to observe NF-κB activation due to heavy ion exposure and to evaluate its potential to lower the detection limit.

### 3.1. NF-κB-Dependent DD-tdTomato Expression after Exposure to Heavy Ions

This study shows that radiation of the same energy (95 MeV/n) and different LET (51 and 272 keV/μm) activates NF-κB signaling differently. NF-κB-dependent DD-tdTomato expression was dose- and LET-dependent, whereby lower doses of heavy ions were required compared to X-rays for activating the genotoxic stress-induced NF-κB pathway. Among heavy ions of different LET, ^36^Ar ions (LET 272 keV/μm) were most effective in activating NF-κB-dependent gene expression in a dose- and LET-dependent manner. NF-κB-dependent reporter gene and target gene expression were previously shown to be LET- and dose-dependent, implicating that the serial events in the NF-κB pathway from DNA binding, transcription, translation and maturation are completed [16,17,18].

It has been a well-known fact that different radiation qualities induce different DNA damage patterns whereby charged particles (heavy ions) compared to photons (X-rays and gamma rays) show increased biological effectiveness due to higher LET [19]. It has be reported that increasing the LET of charged particles increases their lethality due to less effective repair of the resulting clustered DNA damage [20]. When the LET is increased, it affects cells both at the chemical and molecular level and results in complex DNA lesions, DNA strand breaks (SSB, DSB), and activation of DNA damage response pathways. Other studies show that heavy ions with an LET of 34–91 keV/µm are lethal for cells depending on absorbed doses [17]. The maximum killing potential of heavy ions was observed to be ≈100–200 keV/μm, and the maximum RBE for NF-κB activation falls in the same LET range as the RBE of cell killing [18]. The activation of NF-κB and its downstream target genes may provide survival potential to cells through transiently activating genes playing a role in maintenance of DNA fidelity, chromatin remodeling, cytokines expression, histone modification, and cell cycle progression. Other studies also show that the translocation kinetics of the p65 subunit of NF-κB from the cytoplasm to the nucleus depends on radiation quality [21,22,23,24]. A maximal expression of reporter gene after 18 h of radiation was observed due to the accumulation and sustained initiation of reporter gene transcription [9].

### 3.2. Effect of Fluence on NF-κB-Dependent DD-tdTomato Expression

Biological effects in a cell population are not just restricted to the response of individual cells that receive particle hits. Therefore, a collective effect on cell populations was measured in this study. Previously, it was shown that the increase in NF-κB-dependent gene expression is fluence-dependent [16]. In situ studies predicted that low fluences of α particle that even traversed 2% of the nuclei induce gene expression even in non-hit cells [25]. Vares et al. demonstrated that low doses >0.1 Gy of X-rays may trigger adaptive cellular responses in vitro for relatively high doses 1–4 Gy of high LET heavy-ion beams (carbon ions with an LET of 20 and 40 keV/µm and neon ions with an LET of 150 keV/µm) [26]. This pathway is a pro-survival pathway, and it may trigger a radio-adaptive response. In human fibroblasts, low fluences of high-LET heavy ions caused mutations and genomic instability but no difference in cell death [27].

### 3.3. Induction of NF-κB-Dependent DD-tdTomato Expression by a Single Particle Hit

The lowest fluence that was previously shown to activate NF-κB in HEK cells by the reporter d2EGFP was two particles per cell nucleus of argon ions [16]. It was also speculated that the detection of significant NF-κB activation even after only one nuclear traversal of a heavy ion might be possible with an increase in assay sensitivity. Our results depict that ^36^Ar ions activate NF-κB relatively stronger at low fluences compared to ^12^C and ^16^O ions (Figure 3) and that even a single particle hit per nuclear resulted in activation of the NF-κB pathway (Table 1), demonstrating that the ProteoTuner technology increased the sensitivity of the reporter system.

### 3.4. Fold Induction of NF-κB-Dependent Promoter Activity

Shield-1, a small synthetic and membrane permeable molecule, has very high affinity to bind to L106P. The addition of Shield-1 into the medium protects the destabilizing domain linked to the protein of interest from degradation [28]. Another study showed that the ligand is free of off-target effects on gene expression based on microarray data that compared the expression profile of genes in NIH3T3 cells treated with varying concentrations of Shield-1 [29].

Our results indicate that the fluorescence intensity reached higher levels in the presence of Shield-1 compared to cells incubated without Shield-1 after irradiation. This increasing intensity reflects both the stability of protein and also a high promoter activity. When Shield-1 was added into the medium after irradiation, DD-tdTomato accumulated inside the cell. Accumulated fluorescence indicated strong NF-κB activation and provided the evidence that radiation quality based on different LET influences NF-κB activation as determined by its binding to the κB sites in the promoter. The concept that different radiation qualities induce different DNA damage patterns has been proposed and experimentally evaluated for more than three decades [30,31,32,33,34]. However, the current study supports the idea that cellular responses are influenced by LET at different complexity levels including molecular ones even after a single particle hit. This reveals the strong impact that one particle traversal can have on signaling in the hit cell.

## 4. Materials and Methods

### 4.1. Cell Strains and Culture Conditions

The human embryonic kidney cell (HEK/293)-based reporter cell line HEK-pNFκB-DD-tdTomato-C8 already established by Chishti et al. [9] was cultured in minimal essential medium (α-MEM medium, PAN Biotech, Aidenbach, Germany) containing 10% fetal bovine serum (FBS) and incubated at 37 °C, 5%/95% CO_2_/air atmosphere. Cells were counted by an automated cell counter (LUNA, Logos Biosystems, Gyeonggi-do, Korea), and 3 × 10^4^ cells/cm^2^ were seeded into freshly poly-D-lysine (10 µg/cm^2^, Sigma-Aldrich Chemie, Steinheim, Germany) coated culture vessels (25 cm^2^ tissue culture flask, Nunc, Thermo Fisher Scientific Inc., Darmstadt, Germany). Cells were grown for three days and irradiated when they reached the confluency of 50–70%.

### 4.2. Exposure to X-rays

X-rays experiments (LET 0.3–3.0 keV/µm) were performed using the X-ray source RS225 (Gulmay Medical, now: X-Strahl, Surrey, UK) at DLR Cologne. The X-ray tube was adjusted to 200 kV and 15 mA. Using the ionizing chamber TM30013 connected to the dosimeter UNIDOS^webline^ (PTW, Freiburg, Germany), the dose and dose rate were determined. A copper (Cu) filter with a thickness of 0.5 mm was used to eliminate soft X-rays. The dose rate was set to 1.0 Gy/min by adjusting the distance to the X-ray source with an electrically driven exposure table. Samples were irradiated at room temperature. After irradiation, samples were transferred to the incubator and harvested at different time intervals according to experimental requirements.

### 4.3. Exposure to Accelerated Heavy Ions

Exposure to ^36^Ar (95 MeV/n, LET 272 keV/µm), ^12^C (95 MeV/n, LET 73 keV/μm), and ^16^O (95 MeV/n, LET 51 keV/µm) ions was performed at the Grand Accélérateur National d’Ions Lourds (GANIL, Caen, France). The samples were placed in an upright position in the vertical sample holder in front of the beam line. Therefore, the flasks were filled with 50 mL α-MEM-medium without FBS, resulting in ≈1% serum to avoid desiccation. Samples were irradiated at room temperature. Dosimetry was performed by the staff at the accelerator facilities [35], and dose rates were adjusted to ≈1 Gy/min. Fluence was converted to dose by the Equation (1):

(1) $$\mathrm{Dose}\;\left[\mathrm{Gy}\right]=1.6\;\times \;10^{-9}\;\times \;\mathrm{LET}\;\left[\mathrm{keV}/µm\right]\times F\;\left[{P/\mathrm{cm}}^{-2}\right]$$

### 4.4. Treatment with Shield-1

After irradiation, the medium was removed, and freshly prepared medium containing 1 μmol/L Shield-1 (Clontech, now: Takara Bio, Palo Alto, CA, USA) was added to the samples. Cells were incubated in the incubator until harvested for analysis of NF-κB-dependent DD-tdTomato expression.

### 4.5. Formaldehyde Fixation

At defined time points, cells were detached from the flask surface using trypsin (PAN Biotech) and fixed with 3 mL cold 3.7% formaldehyde in phosphate-buffered saline (PBS) prepared by 1:10 dilution of 37% formaldehyde solution (Sigma-Aldrich Chemie) for 30 min at 4 °C. The formaldehyde was diluted with PBS (1:3), and cells were stored at 4 °C. Prior to flow cytometric analysis, cells were centrifuged at 500× *g* for 5 min at room temperature, resuspended in 1 mL PBS, and transferred to flow cytometry round-bottom tubes (BD Falcon, Thermo Fisher Scientific, Schwerte, Germany).

### 4.6. Flow Cytometry

Flow cytometry was performed by fluorescence activated cell scanner (FACScan, Becton Dickinson, now BD Biosciences, San Jose, CA, USA). Forward and side scatter (FSC, SSC) as well as red fluorescence (FL-2) of the samples were measured with an argon laser (488 nm) as an excitation source (Figure 1). In detail, 2 × 10^4^ cells were analyzed at a rate of 200–600 cells per second and depicted using Cell-Quest software (version 1.2, Becton Dickinson). FSC and SSC of the samples were set in a dot plot as a measure of cell size and granularity, and the cell population of interest was gated for further analysis. The DD-tdTomato fluorescence in fluorescence channel FL2 was analyzed using the Flowing software version 2.4.1 (Free online software, Turku Bioscience, Turku, Finland). Using the FL-2 histogram, the extent of NF-κB-dependent DD-tdTomato expression was determined by two approaches: (1) the mean fluorescence of the whole cell population or (2) the percentage of cells with red fluorescence above a threshold. For setting this threshold by means of two markers, untreated and cells treated with a known NF-κB activator were used as follows: DD-tdTomato expressing cells after tumor necrosis factor α (TNF-α) treatment (10 ng/mL) were used as positive control of NF-κB activation and thereby of DD-tdTomato expression (DD-tdTomato(+) cells). Untreated cells expressed only basal levels of DD-tdTomato (DD-tdTomato(–) cells). In the FL-2 histogram, the markers for DD-tdTomato(–) and DD-tdTomato(+) cells were set by means of these untreated and TNF-α-treated cells. The percentage of the cell population displaying fluorescence intensities in the zone of the DD-tdTomato(+) marker was used as a measure of the red fluorescent cell population.

### 4.7. Calculations

#### 4.7.1. Average Hits of Heavy Ions per Cell Nucleus

To calculate the average hits per cell nucleus, the area of the HEK cell nucleus was determined in living bisbenzimide-stained cells (0.5 µmol/L for 45 min). Photographs of the stained nuclei were taken by means of the digital camera Mrx5, which was adapted to the fluorescence microscope Axiovert 135 (Carl Zeiss, Oberkochen, Germany). The nucleus area (A) of HEK cells was measured using the software Axiovision (Carl Zeiss) and yielded an average area of 142 ± 23 µm^2^ [36] The expected fluence (*F_e_*) per cell nucleus was calculated according to Equation (2):

(2) $$F_{e}\;\left[P/\mathrm{cell}\;\mathrm{nucleus}\right]=10^{-8}\;\times \;A\;\left[{µm}^{2}]\times F\;[{P/\mathrm{cm}}^{2}\right]$$

The Poisson distribution of the heavy ion hits in the cell nuclei was calculated according to Equation (3), and the fractions of un-hit and hit cell nuclei were determined.

(3) $$f_{X}\left(x\right)=\frac{F_{e}{}^{x}}{x!}e^{-F_{e}},\;x=\;0,\;1,\;2,\;3,\ldots$$

#### 4.7.2. Fold Induction of Promoter Activity

NF-κB promoter activity was calculated from the mean red fluorescence intensity of reporter cells 18 h after treatment in the presence or absence of Shield-1. The fold induction of NF-κB promoter activity was calculated according to Equation (4).

(4) $$\mathrm{Fold}\;\mathrm{induction}\;=\frac{\mathrm{Fluorescence}\;\mathrm{intensity}\;\mathrm{of}\;\mathrm{cells}\;\mathrm{treated}\;\mathrm{with}\;\mathrm{Shield}\text{-}1\;\mathrm{and}\;\mathrm{inducer}}{\mathrm{Fluorescence}\;\mathrm{intensity}\;\mathrm{of}\;\mathrm{cells}\;\mathrm{treated}\;\mathrm{with}\;\mathrm{Shield}\text{-}1\;\mathrm{alone}}$$

#### 4.7.3. Calculation of Relative Biological Effectiveness for NF-κB Activation

The energy doses eliciting a doubling of DD-tdTomato(+) cells 18 h after irradiation were derived from the dose–effect relationships for the reference radiation (X-rays) and the test radiation (heavy ions). The Relative Biologic Effectiveness (RBE) for NF-κB activation (*RBE*_NF-κB_) was calculated as described previously [13] by using following equation:

(5) $$RBE_{\mathrm{NF}\text{-}\kappa B}=\frac{\mathrm{Energy}\;\mathrm{dose}\;\mathrm{of}\;\mathrm{reference}\;\mathrm{radiation}\;\mathrm{inducing}\;\mathrm{doubling}\;\mathrm{of}\;\mathrm{DD}\text{-}\mathrm{tdTomato}(+)\;\mathrm{cells}\;\left[\mathrm{Gy}\right]}{\mathrm{Energy}\;\mathrm{dose}\;\mathrm{of}\;\mathrm{test}\;\mathrm{radiation}\;\mathrm{inducing}\;\mathrm{doubling}\;\mathrm{of}\;\mathrm{DD}\text{-}\mathrm{tdTomato}(+)\;\mathrm{cells}\;\left[\mathrm{Gy}\right]}$$

### 4.8. Statistics

Each X-rays experiment was repeated at least two times. The beam time is very restricted, and it was not possible to repeat the experiments in independent beam times for every ion analyzed in this work; therefore, three independent biological replicates were used in each experiment with heavy ions. Means and standard errors were calculated with Microsoft^®®^ Office Excel 2010, and graphs were prepared using SigmaPlot 12.0.

## 5. Conclusions

In past few years, there has been an increase in space-related radiation biology research. In the current study, ground-based experiments with high-LET heavy ions were performed with clear evidence that a single particle hit can induce NF-κB-mediated cellular responses. In the presence of Shield-1, the fluorescent protein DD-tdTomato was accumulated inside the cell, which helped to quantify the fold induction of the promoter containing NF-κB-binding sites after heavy ion exposure. Thus, the fluorescent protein DD-tdTomato facilitates the study of NF-κB activation and suppression of its activation. This reporter system represents a powerful approach for fast and convenient NF-κB regulation studies and promisingly contributes to radiation research related to space and predicting tumor response to heavy ion therapy.

Whether the pro-survival effects of NF-κB activation impact human health by contributing to dysplastic changes or even malignancies following long-term space missions during which astronauts were chronically exposed to heavy ions as well as cancer survivors treated with much higher doses of heavy ions during radiotherapy remains elusive. Exposure to ionizing radiation is one of the showstoppers for long-term human interplanetary missions. Knowledge of heavy ion-induced health effects strongly depends on accelerator-based experiments with high-LET heavy ions, and more data are needed to fully assess the risks and to develop suitable countermeasures for deep space travel. Similarly, by combining data on local tumor control with molecular data such as that presented in our study, relevant biomarkers for radiation response may be identified that could be used to predict tumor response to therapy.

## Figures and Tables

**Table 1:** Description of radiation qualities based on energy and linear energy transfer (LET), and dose and average hits per cell nucleus that are required for doubling of NF-κB-dependent reporter gene expression above background.

Radiation Quality	Energy (MeV/n)	Energy on Target (MeV/n) ^1^	LET in H_2_O (keV/µm)	Particles/cm^2^ for 2× NF-κB Activation		Dose for 2× NF-κB Activation ^2^		Average Hits for 2× NF-κB Activation ^3^		RBE_NF-κB_ ^2^	
				- ^4^	Shield-1	-	Shield-1	-	Shield-1	-	Shield-1
X-rays	200 kV		0.3–3	^5^	^5^	~8	~4	^5^	^5^	1	1
^16^O	95	90.6	51	5.00 × 10^7^	N.D. ^6^	~2	N.D.	~44	N.D.	4	N.D.
^12^C	95	28.6	73	3.20 × 10^7^	1.60 × 10^7^	~4	~2	~45	~22	2	2
^36^Ar	95	83.8	272	2.50 × 10^6^	6.25 × 10^5^	~1	~0.25	~3	~1	8	16

^1^ The energy on target was derived from the extraction energy by considering all materials that the beam traversed before it arrived at the plane of the cells, including the bottom of the cell culture vessel. The energy of the carbon ion beam was further reduced by a 16.9 mm poly(methyl methacrylate) PMMA degrader. Calculation of LET is based on the energy on the target. ^2^ The dose for 2× NF-κB activation (D_2_×) is the dose eliciting a doubling of the NF-κB-dependent fluorescence signal. The RBE_NF-κB_ was calculated by dividing D_2_× of X-rays by D_2_× of heavy ions. ^3^ Average hits per cell nucleus and average hits for 2× NF-κB activation were calculated as mentioned in Section 4.7. ^4^ (-) represents experiments without Shield-1. ^5^ As X-rays are not considered to be of particulate nature, the number of hits cannot be calculated. However, it is known that 1 Gy of X-rays results in ≈40 double-strand breaks (DSB)/Gy, 800 single-strand breaks (SSB)/Gy, and even more base damage. ^6^ (N.D.) represents Not Done. Due to some technical and logistic issues, ^16^O ion experiments were not done in the presence of Shield-1.

## Data Availability

Data are shown within the tables and figures of the manuscript and in Supplementary Figure S1.

## Supplementary Materials

The following are available online at https://www.mdpi.com/article/10.3390/ijms222413530/s1.
