# Supplementary material for: The Use of ProteoTuner Technology to Study Nuclear Factor κB Activation by Heavy Ions
Source: Int J Mol Sci. 2021 Dec 16;22(24):13530. doi: 10.3390/ijms222413530 (PMC8703744; doi:10.3390/ijms222413530)
Supplement: Supplementary file 1 [file ijms-22-13530-s001.zip › ijms-1503658-supplementary.pdf]

The use of ProteoTuner technology to study Nuclear Factor κB activation by heavy ions

Arif Ali Chishti, Christa Baumstark-Khan, Hasan Nisar, Yueyuan Hu, Bikash Konda, B. Henschenmacher, Luis F. Spitta, Claudia Schmitz, Sebastian Feles and Christine E. Hellweg

International Journal of Molecular Sciences

Supplementary Material

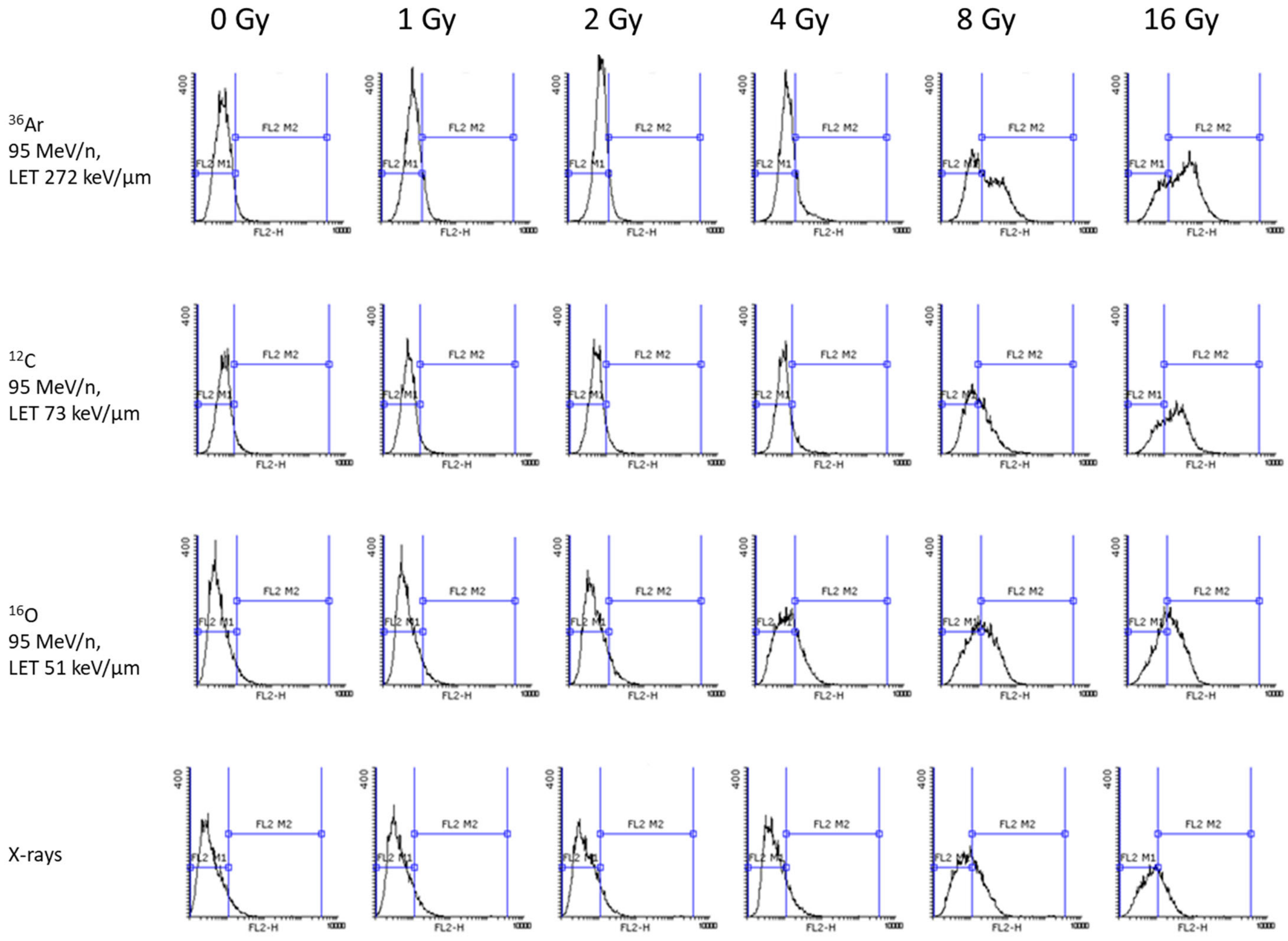

Supplementary Figure S1. Flow cytometry analysis of DD-tdTomato fluorescence. In the histogram, on the X-axis, FL2-H represents height of the red fluorescence signal in channel 2 of the flow cytometer (FL2), and cell counts are displayed on the Y-axis. The population within the range of marker 1 (FL2 M1) in represents cells with background fluorescence and the FL2 M2 population represents cells with NF-κB-dependently induced red fluorescence.
